# SFRP-4 abrogates Wnt-3a-induced β-catenin and Akt/PKB signalling and reverses a Wnt-3a-imposed inhibition of *in vitro *mammary differentiation

**DOI:** 10.1186/1750-2187-3-10

**Published:** 2008-05-02

**Authors:** Thecla Constantinou, Fabrizio Baumann, Markus D Lacher, Susanne Saurer, Robert Friis, Arun Dharmarajan

**Affiliations:** 1 Department of Clinical Research, University of Berne, Tiefenaustr. 120, CH 3004 Berne, Switzerland; 2 Integra Biosciences AG, Schönbühlstrasse 8, CH-7000 Chur, Switzerland; 3 Helen Diller Family Comprehensive Cancer Center, University of California, San Francisco, Dept. of Medicine, Division of Gastroenterology, 2340 Sutter St., N-361, San Francisco, CA 9413-0128, USA; 4 School of Anatomy and Human Biology, University of Western Australia, 35 Stirling Highway, Crawley, Perth, 6009 Western Australia

## Abstract

**Background:**

Conserved Wnt ligands are critical for signalling during development; however, various factors modulate their activity. Among these factors are the Secreted Frizzled-Related Proteins (SFRP). We previously isolated the *SFRP-4 *gene from an involuting rat mammary gland and later showed that transgenic mice inappropriately expressing SFRP-4 during lactation exhibited a high level of apoptosis with reduced survival of progeny.

**Results:**

In order to address the questions related to the mechanism of Wnt signalling and its inhibition by SFRP-4 which we report here, we employed partially-purified Wnt-3a in a co-culture model system. Ectopic expression of SFRP-4 was accomplished by infection with a pBabe^puro ^construct. The co-cultures comprised Line 31E mouse mammary secretory epithelial cells and Line 30F, undifferentiated, fibroblast-like mouse mammary cells. *In vitro *differentiation of such co-cultures can be demonstrated by induction of the *β-casein *gene in response to lactogenic hormones.

We show here that treatment of cells with partially-purified Wnt-3a initiates Dvl-3, Akt/PKB and GSK-3β hyperphosphorylation and β-catenin activation. Furthermore, while up-regulating the *cyclin D1 *and *connexin-43 *genes and elevating transepithelial resistance of Line 31E cell monolayers, Wnt-3a treatment abrogates differentiation of co-cultures in response to the lactogenic hormones prolactin, insulin and glucocorticoid. Cells which express SFRP-4, however, are largely unaffected by Wnt-3a stimulation. Since a physical association between Wnt-3a and SFRP-4 could be demonstrated with immunoprecipitation/Western blotting experiments, this interaction, presumably owing to the Frizzled homology region typical of all SFRPs, explains the refractory response to Wnt-3a which was observed.

**Conclusion:**

This study demonstrates that Wnt-3a treatment activates the Wnt signalling pathway and interferes with *in vitro *differentiation of mammary co-cultures to β-casein production in response to lactogenic hormones. Similarly, in another measure of differentiation, following Wnt-3a treatment mammary epithelial cells could be shown to up-regulate the *cyclin D1 *and *connexin-43 *genes while phenotypically they show increased transepithelial resistance across the cell monolayer. All these behavioural changes can be blocked in mammary epithelial cells expressing SFRP-4. Thus, our data illustrate in an *in vitro *model a mechanism by which SFRP-4 can modulate a differentiation response to Wnt-3a.

## Background

The mammary gland, with each oestrus cycle, undergoes a round of proliferation and partial differentiation followed by apoptosis of a fraction of the terminally-differentiated secretory cells [1-3]. Some years ago we developed the Lines 31E-30F co-culture model for the study of differentiated functions of mammary epithelial cells responding to the lactogenic hormones insulin, glucocorticoid and prolactin by inducing transcription and secretion of the milk protein β-casein [4,5].

Signalling by the Wnt family of secreted glycoprotein ligands is essential both in embryonic [6,7] as well as adult development, where they are implicated in the maintenance of stem cell pools [8-12], in tissue differentiation programs [11-13] and in cell survival [13,14]. It is, therefore, unsurprising that the pathogenesis of a variety of diseases, including cancer [11,12,15-17], frequently reflects disturbances in Wnt signalling.

Although Wnt proteins are secreted, the difficulty in solubilising active Wnt molecules hindered attempts to purify the Wnts and delayed a thorough biochemical characterisation of this growth factor family. Willert et al. [8] recently isolated a Wnt-3a-secreting L cell line and developed a purification protocol allowing Wnt-3a to be obtained from cell culture supernatants in a biologically active and relatively pure form. The insoluble nature of Wnts was explained by the observation that these proteins are palmitoylated and, thus, more hydrophobic than was initially predicted from the primary amino acid sequence [8].

Genetic and biochemical data have demonstrated that the seven-pass transmembrane Frizzled proteins (Fz) are the receptors for the Wnts [18,19]. The Frizzled family consists of at least 10 mammalian members and is named after the *Drosophila *tissue polarity gene, *frizzled *(*fz*). All members of the Fz family expose an N-terminal extracellular sequence designated the Cysteine-Rich Domain (CRD), consisting of 120 amino acids including 10 conserved cysteines, which is able to bind Wnt ligands with nanomolar affinity [20,21]. Wnt binding, however, also requires the presence of a co-receptor, a single-pass transmembrane molecule of the LRP (Low Density Lipoprotein Receptor-related Protein) family, either LRP-5 or -6 in vertebrates [22-24]. Wnts bind to LRPs and Fz to form a receptor trimeric complex [23].

Wnt signals are transduced through at least three intracellular signalling pathways which cross-regulate one another as well as interacting with other signalling networks [25-27]. These are: the Wnt/Ca^2+ ^pathway, the planar cell polarity pathway (PCP) and the so-called canonical Wnt/β-catenin pathway. The Wnt/Ca^2+ ^pathway involves the activation of phospholipase C (PLC) and leads to elevated Ca^2+ ^levels and the activation of protein kinase C [28,29]. The PCP pathway acts through small GTPases such as RhoA and Rac and activated c-Jun amino-terminal kinase (JNK) [30]. For the canonical Wnt pathway, β-catenin ultimately participates in a co-activator complex in the nucleus with transcription factors of the TCF/LEF family and several other proteins which regulates target gene transcription [27,31]. Interestingly, all three signalling pathways apparently utilize Dishevelled (Dsh in Drosophila and Dvl in mammals) as a signalling intermediate [26].

In addition to the requirement for an LRP-5/6 co-receptor, Wnt signalling is modulated extracellularly by diverse secreted proteins, including members of the Dickkopf (Dkk) and Secreted Frizzled-related Protein (SFRP or FrzB) families. Dkk proteins, whose expression is regulated by certain Wnts, do not themselves bind Wnts, but instead interact with the extracellular domain of the LRPs, thereby denying activation by Wnts [32,33]. On the other hand, SFRPs can bind to Wnts by virtue of their CRD, which is strikingly homologous to that of the Fz receptors, but lacks any transmembrane domain [25,34-38]. Hence, as competitors for Wnts, SFRPs can interfere with Wnt-Fz signalling, but they can also interact directly with the Fz receptors themselves [39]. Hence, a second way in which SFRPs might antagonise Wnt signalling is through the formation of a non-functional complex with Frizzled receptors; however, it has not been ruled out that depending on expression levels or cellular context, SFRPs could promote Wnt signalling either by protecting Wnts from degradation or by facilitating Wnt secretion/transport [40].

SFRP-4 was first isolated in our laboratory [36,41] from a rat ovarian cDNA library (Accession number EMBL: AF012891) [36]. We subsequently prepared transgenic mouse lines inappropriately expressing rat SFRP-4 in the mammary gland during pregnancy and lactation [42]. With these lines, we showed that SFRP-4 expression was associated with apoptosis in mammary secretory epithelial cells, partly compensated by concomitant increased cell proliferation, but still resulting in lactational insufficiency [42].

In the present communication, we report the effects of treatment with partially-purified Wnt-3a on canonical Wnt signalling and the impact of ectopic SFRP-4 expression on signalling intermediates such as β-catenin and Dishevelled (Dvl) and on downstream Wnt "target gene" expression. Building on these results, we then investigated the phenotypes produced by Wnt-3a with and without ectopic SFRP-4 expression in epithelial cell polarisation and with the differentiated response in the Line 31E+30F mouse mammary co-culture model following lactogenic hormone treatment [4,5].

## Results

Because SFRP-4 is strongly upregulated during involution of the mouse mammary gland and is otherwise virtually absent from this tissue, we wanted to develop a simplified mammary model allowing the study of its effects on Wnt signalling *in vitro*. In order to be able to study the effects of ectopically-expressed SFRP-4, we used partially-purified Wnt-3a prepared by a convenient protocol based on that described by Willert et al. [8]. To study the phenotype following Wnt-3a treatment and the impact of SFRP-4 on this phenotype, we employed the Lines 31E (epithelial) +30F (fibroblast-like) co-culture model previously developed in our laboratory [4,5]. Co-cultures respond to lactogenic hormone treatment by synthesis and secretion into the medium of β-casein.

To define the response of these cells to purified Wnt-3a, we monitored several steps in Wnt signalling pathways: two parameters used for the Wnt-3a mediated signalling were hyperphosphorylation of Dishevelled [43,44] and levels of free cytosolic, or "active" β-catenin [45]. Other downstream cytoplasmic signalling intermediates, GSK3β and Akt/PKB [42,46,47] were also evaluated using phospho-specific antibodies on immunoblots. Finally, the measure of full pathway activation was the expression of two known Wnt target genes, *cyclin D1 *[48] and *connexin-43 *[49].

### Phosphorylation of Dvl

**Figure 1:**
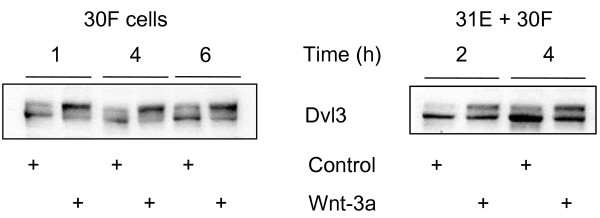
**Dvl-3 as a marker of Wnt pathway activation**. Line 30F (left panel) or co-cultures of Lines 31E + 30F (right panel) stimulated with ectopic Wnt-3a respond with hyperphosphorylation of Dvl-3. Following treatment with Wnt-3a for the indicated times, cells were lysed with Triton X-100 Lysis Buffer and cell extracts were subjected to Western blot analysis. Hyperphosphorylation of Dvl-3 is evident by a consistent relative increase in the intensity of the upper band of the doublet. Alkaline phosphatase treatment caused the loss of the upper band (data not shown).

Previous studies have shown that Dvl undergoes hyperphosphorylation in response to Wnt signals that stabilise β-catenin (44; T. Constantinou, unpublished). Among the Dvl family, Dvl-3 has recently been shown to undergo the greatest proportional transition from the cytoplasmic to the plasma membrane compartment after Wnt-3a stimulation [50]. This presumably correlates with phosphorylation status. Thus, we analysed the effect of Wnt-3a stimulation on the phosphorylation status of Dvl-3 in our cells, using a polyclonal antibody specific for Dvl-3 which was prepared in our laboratory as described in Materials and Methods. As shown in Fig. 1, Dvl-3 could be detected with immunoblots as a double band with a molecular mass of approximately 90 kDa. This double band consists of hypophosphorylated (lower band) and hyperphosphorylated (upper band) Dvl-3. In 30F cells untreated with Wnt-3a, the lower band is more intense while the upper band is faint and diffuse. Wnt-3a stimulation results in an intense, distinct upper band and a fainter lower band. These differences are also observed in Lines 31E + 30F (4:1) co-cultures (Fig. 1).

### Accumulation of free β-catenin in the cytoplasm

**Figure 2:**
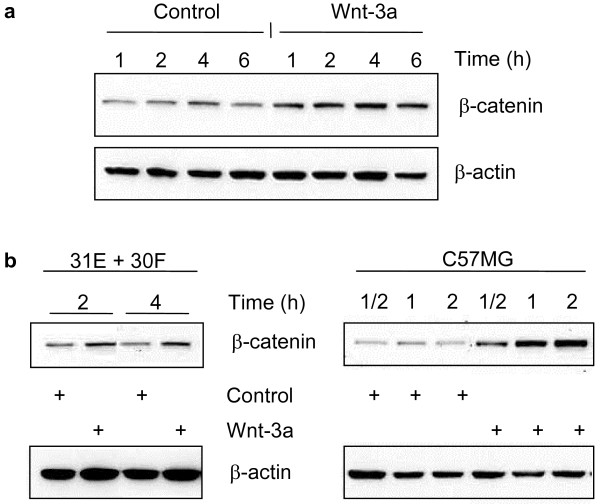
**Accumulation of β-catenin in the cytosol upon stimulation with Wnt-3a**. Line 30F cells **(a)**, and co-cultures of Lines 31E + 30F or C57MG cells **(b) **were stimulated with purified Wnt-3a or mock-purified conditioned medium (Control) for the indicated times. Free cytoplasmic β-catenin was obtained by hypotonic lysis using Dounce homogenization followed by immunoblot analysis with a broadly reactive polyclonal, anti-β-catenin antibody. Increased "free" cytosolic β-catenin is evident even after half an hour of Wnt-3a treatment of C57MG cells **(b)**, and is sustained for up to the 6 hour time point examined with 30F cells **(a)**. Immunodetection of β-actin served as a loading control.

**Figure 4:**
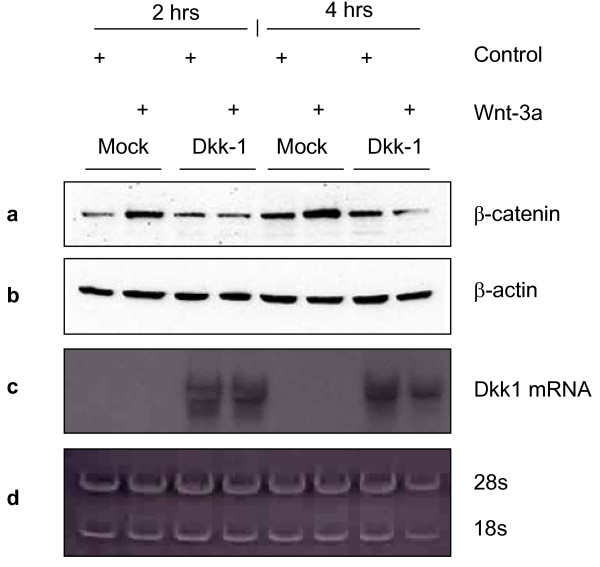
**Inhibition of Wnt-3a pathway activation by Dkk-1**. L cells were transiently transfected with an empty vector (Mock) or with a plasmid encoding Dkk-1 and were either control-treated or purified Wnt-3a-treated for 2 and 4 hours. **(a) **At both time points cells transfected with the Dkk-1 plasmid fail to induce accumulation of cytoplasmic β-catenin upon Wnt-3a treatment, as detected by Western Blot. In contrast, Mock transfected cells show an elevation in the level of β-catenin upon Wnt-3a treatment. **(b) **The detection of β-actin served as a loading control. **(c) **Parallel plates were treated as above and lysates were collected for extraction of total RNA. *Dkk-1 *mRNA is shown by Northern Blot in the cells transfected with Dkk-1. **(d) **18S and 28S rRNA bands are loading controls obtained by acridine orange staining of the gel used for the Northern Blot prior to blotting.

**Figure 5:**
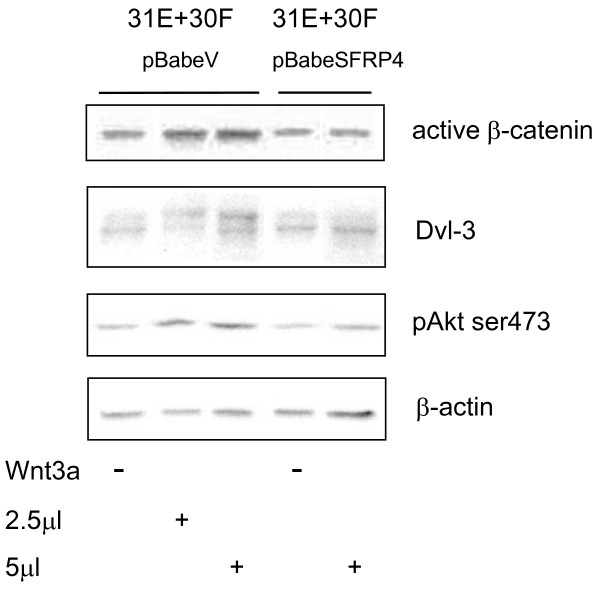
**SFRP-4 interferes with Wnt-3a signalling in Lines 31E + 30F co-cultures**. pBabeSFRP-4-infected, puromycin-selected, 31E cells in co-culture with 30F cells fail to show Wnt and Akt signalling activation by Wnt-3a. Treatment with Wnt-3a induced a hyperphosphorylation of Dvl-3, an increase in Akt phosphorylation (Ser473), and raised the levels of "active" β-catenin in the absence (pBabeV, vector), but not in the presence of the SFRP-4 cells.

**Figure 8:**
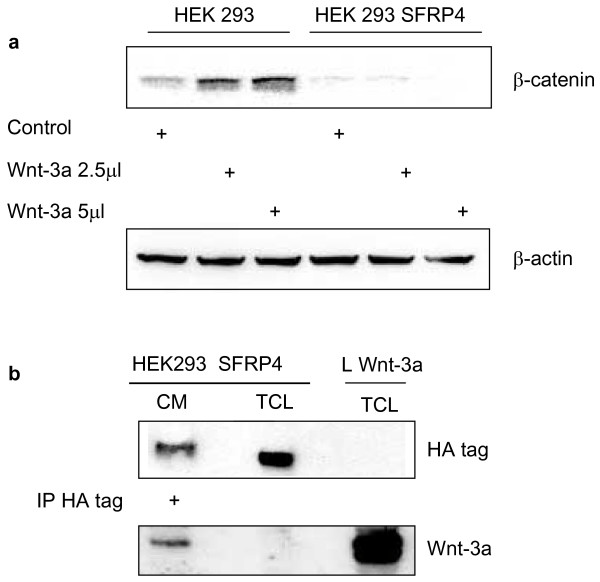
**SFRP-4 binds to Wnt-3a, and thereby prevents activation of the β-catenin pathway**. **(a) **HEK293 and HEK293 transfected with SFRP-4(HA tagged) were either control-treated or purified Wnt-3a-treated for 4 hours. Cytosolic lysates were analysed by Western Blot for the levels of β-catenin. Pathway activation is indicated by the increase in cytosolic β-catenin upon Wnt-3a treatment seen only in the parental HEK293 cells whereas in the SFPR-4-expressing cells this effect was abolished. **(b) **conditioned medium supernatant (CM) prepared from the HEK 293 cells expressing SFRP-4(HA tagged) after treatment with 5 μl Wnt-3a for 4 hours in the above experiment, was immunoprecipitated with an anti-HA-tag antibody, electrophoresed, Western blotted and, firstly, detected with an anti-HA-tag antibody. The first lane shows the precipitated SFRP-4 containing the HA tag from the CM. The middle lane shows the presence of the same protein in the total cell lysate (TCL) of the same cells as a control. The membrane was then re-probed with affinity-purified anti-Wnt-3a antibody, showing binding of Wnt-3a to the immunoprecipitated SFRP-4(HA tagged) from the conditioned medium supernatant (first lane), but not in the total cell lysate (middle lane). In addition, a sample of TCL from L cells expressing Wnt-3a (Fig. 8b, last lane), which had not been subjected to immunoprecipitation, served as a control demonstrating the detection of Wnt-3a protein.

Stabilisation and accumulation of β-catenin in the cytoplasm is a hallmark of canonical Wnt signalling and thus we used β-catenin accumulation in the cytoplasm as a prime indicator for pathway activation. The pool of β-catenin associated with membrane E-cadherin is much larger than the relatively small pool of free β-catenin in the cytoplasm. Even following stimulation with a Wnt ligand with concomitant increase in the cytosolic β-catenin, if one examines a total cell lysate, cytosolic β-catenin is masked by this large amount of β-catenin present at the membrane. Hence, we analyzed the fraction of free, cytosolic β-catenin in two ways, i) with cytosolic lysates obtained after Dounce homogenization of cells swollen in hypotonic buffer, and, ii) with lysates obtained by Triton X-100-containing buffers. Detection of the relative amounts was with immunoblots, using a "pan" β-catenin-specific antibody for the cytosolic lysates (Figs. 2, 4a, 8a), while for Triton X-100 cell lysates (Fig. 5) detection was with a monoclonal antibody developed by van der Noort et al. [45] to detect "active" β-catenin lacking the N-terminal phosphorylation responsible to its association with the axin/APC/glycogen synthetase kinase-3β destruction complex. Both procedures demonstrated the same effects following Wnt-3a treatment.

For the characterisation of the time-dependent response to purified Wnt-3a on β-catenin turn-over, mammary cell lines were treated with 5 μl of the partially-purified Wnt-3a for several time points (Fig. 2) and analysed using cytosolic lysates. 30F cells were treated for 1, 2, 4 and 6 hours with Wnt-3a and compared to the control untreated cells, showing an increase in free, cytosolic β-catenin that is sustained up to the 6 hour treatment (Fig. 2a). The co-cultures of Lines 31E + 30F cells (in proportion 4:1) were analysed for 2 and 4 hours. Fig. 2b shows that co-cultured cells responded similarly to the treatment, while the C57 normal Mammary Gland cell line (C57MG) exhibited a higher differential response in β-catenin accumulation owing to lower basal levels of cytosolic β-catenin. When C57MG cells were treated for half an hour, 1 and 2 hours, cytosolic β-catenin increased in a time dependent manner (Fig. 2b).

### Phosphorylation of Akt and GSK3β

**Figure 3:**
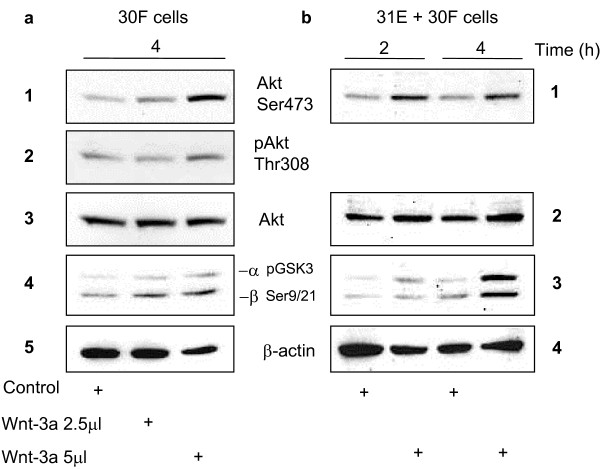
**Wnt signalling induces phosphorylation and activation of Akt/PKB**. **(a) **Upon stimulation with 5 μl/ml of purified Wnt-3a for 4 hours, Line 30F cells show an elevation of phoshorylated Akt in both Ser473 (panel 1) and Thr308 (panel 2) which coincides with phosphorylation of GSK3β at Ser9/21 (panel 4), reflecting its inactivation by Akt. Total Akt was used as a loading control by stripping the membrane and reprobing with an anti-Akt antibody (panel 3). β-actin was used as a loading control for pGSK-3β (panel 5). **(b) **Treatment of 31E + 30F co-cultures with purified Wnt-3a for 2 and 4 hours also resulted in phosphorylation of Akt at Ser473 (panel 1) and of GSK-3α and -3β at Ser9/21 (panel 3).

Stimulation of Line 30F or co-cultured 31E + 30F cells with Wnt-3a for 2 and 4 hours resulted in the phosphorylation of Akt at both Ser473 and (weakly) Thr308 in Triton X-100 lysates (Fig. 3a, panels 1 and 2). Accordingly, Akt activity, measured by its ability to phosphorylate GSK3β at Ser9 with attendant inactivation, was higher in Wnt-3a-treated than in the control cells (Fig. 3a, panel 4 and Fig. 3b, panel 3). These results show that phosphorylation of both Akt and GSK3β are responses to Wnt-3a stimulation. The same lysates from the co-culture experiment had been analysed for Dvl-3 hyperphosphorylation following Wnt-3a treatment (data shown in Fig. 1). Akt did not interact with Dvl-3 directly as confirmed by co-immunoprecipitation assays (data not shown). These results, however, are consistent with the existence of the postulated cross-talk between Wnt and Akt signalling [42,46].

### Effect of a Wnt Inhibitor, Dickkopf

Modulation of a Wnt-transmitted signal can be controlled by various inhibitors acting extracellularly, in the cytoplasm, or in the nucleus. We were interested in secreted extracellular inhibitors of Wnt signalling and, more specifically, in SFRP-4. We wanted to establish whether SFRP-4 produces a phenotype in mammary cells and whether its function is through binding to the Wnt-3a ligand, or through some other mechanism. Dkk-1, a well-characterized inhibitor of the Wnt pathway acting in a ligand-independent manner was investigated as a control.

Dkk-1 is a secreted antagonist of Wnt/β-catenin signalling that acts by direct binding to the Wnt co-receptor LRP5/6 and with Kremen in a ternary complex triggering internalisation and clearance of LRP5/6 from the cell surface [32,33]. Thus, Dkk-1 is appropriate for our purpose when studying Wnt pathway inhibition as it does not depend on the presence of a particular Wnt ligand for its mode of action, but instead makes the co-receptor unavailable. To test this, because of their highly efficient transfection, L cells were transiently transfected with either Dkk-1 or an empty (Mock) vector and were then treated with purified Wnt-3a for 2 and 4 hours. The effect of Dkk-1 was assessed by analysing the amount of free, cytosolic β-catenin. In Mock-transfected cells, the amount of β-catenin increases following Wnt-3a treatment as compared to the control treated at both time points (Fig. 4, lanes 1, 2, 5 and 6). Dkk-1 transfected cells, however, fail to induce an increase in cytosolic β-catenin following stimulation with Wnt-3a (Fig. 4, lanes 3, 4, 7 and 8). This result is in agreement with the known mode of action of Dkk-1 [23,32].

### Inhibition of Wnt-3a Signalling in Mammary Cells which are expressing SFRP-4

The hypothesis that SFRPs compete with Fz receptors for Wnt ligands has been widely discussed and an inhibitory activity has been established for SFRP-1 and -2 against Wnt-3a [51]. Fig. 5 illustrates the response to Wnt-3a treatment of co-cultured cells where Line 31E cells had been infected with either pBabe^puro ^vector or pBabe^puro ^SFRP-4 followed by puromycin selection. In Fig. 5, immunoblots are presented in which Triton X-100 lysates were analysed using a monoclonal antibody specifically reactive with "active", cytosolic β-catenin [45]. Elevated "active" β-catenin levels, hyperphosphorylation of Dvl-3 and increased levels of phospho (Ser 473) Akt were apparent where the "empty" retroviral vector was employed. Wnt-3a stimulation of cells infected with pBabe^puro ^SFRP-4, however, failed to cause elevated levels of "active", cytosolic β-catenin. Similarly, Line 31E SFRP-4 cells in co-culture showed no changes in Dvl-3 or phospho (Ser473)-Akt in response to Wnt-3a. Hence, an inhibitory effect of SFRP-4 expression on Wnt-3a stimulation was apparent.

### Influence of SFRP-4 on Wnt-dependent Gene Expression

**Figure 6:**
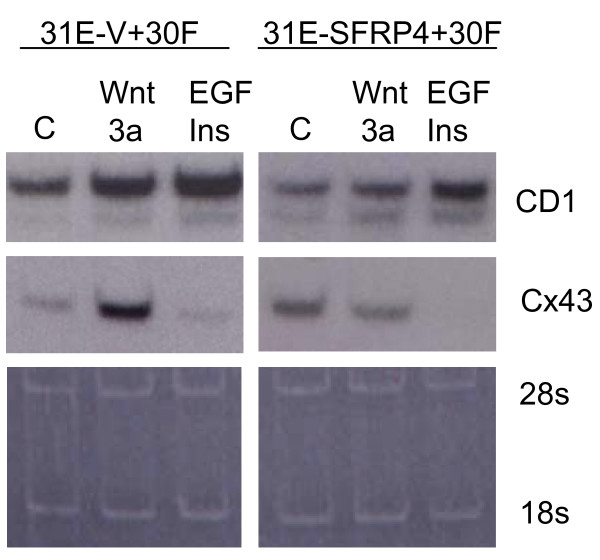
***Cyclin D1 *and connexin-43 mRNA expression in Lines 31E + 30F co-cultures after Wnt-3a stimulation**. Northern blots were employed to show the expression of *cyclin D1 *and *connexin-43 *polyA^+ ^RNAs after stimulation of the cultures for 12 hours with Wnt-3a or insulin + epidermal growth factor (EGF). Co-cultures, made with either pBabeVector or pBabeSFRP-4-infected Line 31E cells together with Line 30F cells, were maintained in 3% serum-containing medium and compared in their responses to Wnt-3a. With Line 31E-Vector cells (31E-V) in co-culture, either Wnt-3a alone or insulin + EGF (Ins+EGF) treatments elicit stimulation of *cyclin D1 *(CD1) mRNA expression, whereas *connexin-43 *(Cx43) expression responds only to Wnt-3a, and is strongly inhibited by EGF+insulin. A response to Wnt-3a was not seen; however, when co-cultures were made using Line 31E cells expressing pBabeSFRP-4 (31E-SFRP4), up-regulation of *connexin-43 *but not *cyclin D1 *was prevented. However, *cyclin D1 *mRNA up-regulation is reduced compared to the co-cultures containing Line 31E-Vector cells. Residual ribosomal RNA (loading control) was stained with acridine orange.

The canonical Wnt signalling pathway leads to increased nuclear localization of β-catenin as a complex with the TCF/LEF family of transcription factors and to changes in regulation of gene expression for known "target" genes [19]. In Fig. 6, Northern blots show responses by 31E+30F co-cultures to Wnt-3a or epidermal growth factor (EGF)+insulin stimulation. The *cyclin D1 *and *connexin-43 *genes are two recognized targets of stimulation by various Wnt ligands including Wnt-3a [48,49]. Co-cultures in which the Line 31E component had been previously infected with pBabe^puro^SFRP-4 followed by puromycin selection failed to exhibit up-regulation of *connexin-43 *expression in response to Wnt-3a. Interestingly, *cyclin D1 *expression was still induced by Wnt-3a; however, the extent of up-regulation was reduced in the presence of the 31E-SFRP4 cells.

### Impact of Wnt-3a and SFRP-4 on Differentiated Function of Mammary Co-cultures

A functional assay for the effect of Wnt-3a on Line 31E cells was developed based on its ability to cause increased transmonolayer electrical resistance on the polarized, normal mouse epithelial cells [5]. Resistance measurements were obtained with the Millipore (Bedford, MA., USA) Millicell-ERS instrument, essentially a milli-volt/ohmmeter that provides an alternating voltage source to minimize cell damage. Increased transmonolayer resistance 24 hours following Wnt-3a stimulation (Table 1) is presumably a consequence of upregulation of proteins responsible for cell: cell adhesion [52]. Table 1 shows that ectopic SFRP-4 expression in Line 31E cells prevents the increased transmonolayer resistance exhibited by control Line 31E cells in response to treatment with purified Wnt-3a.

**Table 1:** Electrical resistance across monolayers of cultured Line 31E mammary epithelial cells

**Cell Type**	**Treatment**	Transmonolayer resistance* (Ohms × cm^2^**)**
31E-pBabeVector	w/o Wnt-3a	290 +/- 22
	+ Wnt-3a	620 +/- 36
31E-pBabeSFRP-4	w/o Wnt-3a	250 +/- 18
	+ Wnt-3a	310 +/- 27

* Cultures previously selected with puromycin were grown on nitrocellulose filter inserts (2.2 cm diameter) for 48 hrs prior to adding treatment with or without Wnt-3a at 5 μl/ml for a further 28 hrs. Values shown are the mean +/- S.E.M. of 5 measurements made on parallel inserts taken across the membrane from apical to basal sides with the Millicell ERS instrument.

**Figure 7:**
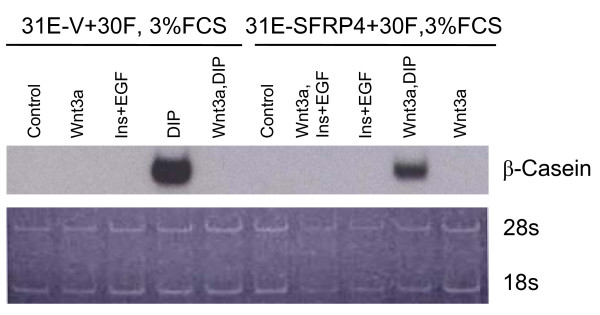
**The Wnt-3a-imposed abrogation of differentiated response to lactogenic hormones was mitigated in pBabeSFRP-4-infected Line 31E cells**. Using Northern blot methods, the expression of β-casein was investigated after stimulation of cultures with Wnt-3a and/or different combinations of growth factors and lactogenic hormones. Lines 31E +30F co-cultures containing either pBabeVector or pBabeSFRP-4-infected Line 31E cells were compared. Most notable is that Wnt-3a is capable of completely suppressing the differentiation to β-casein expression stimulated by dexamethazone-insulin-prolactin (DIP). However, co-cultures containing Line 31E cells infected with SFRP-4 show definite β-casein induction in spite of Wnt-3a treatment. Ribosomal RNA was stained with acridine orange as a loading control.

β-casein expression is a marker for differentiated, functional secretory mammary epithelial cells. This response can be elicited *in vitro *with 31E+30F co-cultures (4:1) following treatment with the lactogenic hormones insulin, glucocorticoid and prolactin. Wnt-3a treatment quantitatively inhibits this differentiated response (Fig. 7); however, co-cultures made with 31E cells stably expressing SFRP-4 are able to respond functionally to lactogenic hormone treatment, presumably because, as shown above, they show a diminished signalling response to Wnt-3a.

### Physical Association of Wnt-3a with SFRP-4

It is reasonable to assume that SFRP-4 might exert its effect by competing with Fz for the Wnt ligand. Indeed, a physical association has been shown *in vitro *for several SFRPs with Wnts [38]. We, therefore, investigated the possible interaction between Wnt-3a and SFRP-4. To perform this experiment, HEK293 cells were stably transfected with an SFRP-4 plasmid construct tagged with a triple influenza hemagglutinin (HA tagged) motif. These cells were then treated with Wnt-3a for 4 hours. Cytosolic lysates were collected and analysed for the level of β-catenin using a Western Blot procedure. The parental HEK293 cells exhibited stimulation after treatment with two different amounts of purified Wnt-3a as shown by the elevation of cytosolic β-catenin (Fig. 8a, lanes 2 and 3). In contrast, HEK293 cells expressing SFRP-4(HA tagged) failed to accumulate β-catenin in the cytosol upon Wnt-3a treatment (Fig. 8a, lanes 5 and 6). Conditioned medium (CM) from HEK293 SFRP-4(HA tagged) cells treated with 5 μl/ml of purified Wnt-3a for 4 hours was saved and used to investigate whether SFRP-4 and Wnt-3a formed complexes. This was done with an immunoprecipitation/Western blot experiment. Supernatant medium was incubated with a rabbit anti-HA tag-specific antibody, complexes isolated, electrophoresed and immunoblotted. Detection was performed, firstly, using a mouse anti-HA tag antibody. Fig. 8b shows the presence of immunoprecipitated SFRP-4(HA tagged) which had been secreted into the medium (Fig. 8b, first lane). SFRP-4(HA tagged) from a total cell lysate (TCL) made with a parallel plate is shown in Fig. 8b (middle lane). There is some difference in the mobility of the two proteins; one explanation could be further post translational modification (maturation) of the secreted, free SFRP-4. The same blot was then re-probed with anti-Wnt-3a antibody revealing that Wnt-3a apparently does co-precipitate with SFRP-4 secreted into the CM (Fig. 8b (first lane), but is not observed with the band from the TCL (middle lane). The last lane (Fig. 8b) was loaded with a total cell lysate from Wnt-3a-producing L cells as a control.

## Discussion

As with many developmental genes, it was the recognition that tumours were caused by inappropriate Wnt-1 expression in many cases of mouse mammary tumour virus disease which focused attention on the fundamental role of Wnts in development, control of cell proliferation and differentiation [6]. The canonical Wnt pathway exhibits so many facets because it is itself responsive to a great many stimulatory and inhibitory signals. Not only are there multiple Wnt ligands acting on multiple Fz receptors [29] as modulated by the competitive, inhibitory activities of the SFRPs [25], but there is the dependence on co-receptor function from the LRPs, again as influenced by expression the Dkk proteins [27,32]. In this complex system, it seems certain that the ultimate developmental message decoded in different target cell populations can be quite different according to differentiation status, and perhaps, even the nutritional conditions in which the recipients find themselves. At the very least, other signalling pathways will contribute nuances to the decoded message [29].

The history of the Wnts in mammals began with mammary tumours; thus, it seemed appropriate to study a mammary model. Our goal in this study was to examine the role of Wnt signalling in affecting mammary cell proliferation and differentiated function, and then to ask, how SFRP-4 would modify the message and change the resulting cell phenotype. At the same time, it was desirable to present a Wnt ligand which was biologically active and partially purified to be relatively free of other factors which could complicate the analysis. This requirement was met by taking advantage of the L cell system secreting soluble, active Wnt-3a, and a simplified purification protocol [8].

As first step we characterized the mammary cells and co-culture model with respect to the Wnt-3a response in a signalling pathway. The co-culture model comprises two cell lines: Line 31E, a polarized epithelial cell exhibiting a high transmonolayer resistance, and forming typical "domes" on plastic substrates [5], and Line 30F, a fibroblast-like, undifferentiated cell type, lacking the characteristics of polarization and expressing vimentin. For optimal lactogenic hormone response, Line 31E cells must be plated with Line 30F cells, which apparently contribute to the formation of "organoid-like" structures [4]. Following dexamethazone, insulin and prolactin treatment, co-cultured cells express β-casein mRNA and secrete β-casein protein [5].

We monitored the canonical pathway of response to Wnt-3a treatment by following Dvl-3 hyperphosphorylation and levels of free, cytosolic β-catenin in Western blots. Global Dvl-1, -2, and -3 phosphorylation has been implicated as an activating step that allows Dvl to signal downstream to β-catenin [27,44]. Experiments shown in Figs. 1 and 2 illustrate these responses to purified Wnt-3a. Dvl-3 hyper-phosphorylation and elevated levels of cytosolic β-catenin could be detected within 1/2 to 1 hour and was maintained up to at least 6 hours. Furthermore, as shown in Fig. 3, Wnt-3a stimulation leads to increased phosphorylation of Akt/PKB at Ser473 and slightly at Thr308. A parallel hyperphosphorylation in Glycogen Synthase Kinases (GSK-3α and -3β) was also observed, both in Line 30 F cells alone, and in the co-cultures of 31E + 30F. Phosphorylation of both Akt and GSK3β are responses to Wnt-3a stimulation [42,47]. These same lysates from the co-culture experiment had been analysed for Dvl-3 hyperphosphorylation following Wnt-3a treatment (shown in Fig. 1). This probably explains why other factors, such as insulin and EGF, that stimulate Akt through the PI3K pathway, but do not activate Dvl, fail to cause an increase in cytosolic β-catenin. Akt did not interact with Dvl-3 directly as confirmed by co-immunoprecipitation (data not shown) and all these results indicate the existence of postulated cross-talk between Wnt and Akt signalling [46] although the mechanism involved needs to be confirmed. We postulate that Dvl interaction with Axin induces a conformational change, allowing Axin-bound GSK3β to be phosphorylated by Akt [42,46]. This phosphorylated GSK3β cannot modify β-catenin efficiently and this results in accumulation in the cytosol. Independent of the exact mechanism involved, the observed phosphorylation of Akt as a consequence of Wnt stimulation is relevant for explaining the anti-apoptotic role of Wnts as survival factors [42-47].

With the goal of establishing the possible nature of an inhibitory activity of SFRP-4 on Wnt-3a stimulation, we used Dickkopf as a control, an inhibitor known to interfere with all Wnt signalling by virtue of competing for the essential co-receptors, LRP-5 or -6. Fig. 4 illustrates this inhibition of Wnt-3a stimulation using increased cytosolic β-catenin as a marker. Using as a measure the "active" β-catenin levels [45] and Dvl-3 hyperphosphorylation as parameters, Fig. 5 demonstrates that SFRP-4-infected co-cultured cells are also inhibited in their responses to Wnt-3a.

The end effect of Wnt-3a stimulation of mammary cells is a phenotypic change conferring *in vitro *corresponding changes in cell proliferation and in cell biological behaviour. Monitoring the expression of two known target genes of Wnt signalling, we could show that both Wnt-3a and insulin/EGF treatments cause an up-regulation in cyclin D1 mRNA expression (Fig. 6). Connexin-43 expression does not respond to Insulin/EGF, but is up-regulated by Wnt-3a. When co-cultures containing Line 31E cells infected with pBabeSFRP-4 were employed for the same experiment, the stimulatory activity of Wnt-3a was reduced, although the Insulin/EGF effect was unchanged (Fig. 6).

Most dramatic was the differentiated response of co-cultures in Fig. 7 after treatment with dexamethazone, insulin and prolactin (DIP). β-casein mRNA expression was completely abrogated by Wnt-3a treatment of co-cultures containing Line 31 E cells infected with pBabe Vector (Fig. 7). Co-cultures containing pBabe SFRP-4-infected Line 31E cells, however, showed β-casein expression only slightly reduced by Wnt-3a treatment. The fact that the results were less clear-cut for cyclin D1 and connexin-43 (Fig. 6) is explainable because the 20% of the cell population made up of Line 30F cells is entirely unable to make β-casein and thus makes no contribution (Fig. 7), whereas Line 30F cells do respond to Wnt-3a for cyclin D1 and connexin-43. Thus, SFRP-4 seems unable to function "para" in these co-cultures.

It has been widely postulated that the effect of SFRP-4 on Wnt stimulation is a consequence of competition with Fz for ligand availability [25,38]. Using an immunoprecipitation/Western blotting experiment similar to that of Wawrzak et al. [38], the results in Fig. 8 show that SFRP-4, tagged for convenience with an HA marker, physically associates with Wnt-3a. HEK293 cells were employed for this experiment, because of the better transfection efficiency and SFRP-4 secretion/release.

Our results present a coherent view of the role of Wnt stimulation in behavioural changes in mammary epithelial cells, and how SFRPs can modulate such changes. Multiplied by the variety of Wnt, Fz and SFRP players, it is possible to see, how even just within this organ, complex behavioural permutations could be achieved. One feature of the Wnts, their insolubility and association with extracellular matrix components, provokes speculation that their action *in vivo *will be long-lasting in fields surrounding the producing cells, presumably in a concentration gradient. In fact, numerous *in vivo *studies address the role of Wnt signalling in different stem cell populations [8-11], and in various developmental stages of mammary development [12]. It is an interesting feature of SFRPs, being as they are, secreted, and responding rapidly to transcriptional regulation [53], that they offer a mechanism for saturating and neutralizing long-lasting extracellular matrix-bound Wnt-activity fields. Since from the outset of work with SFRP-4 [41], its role seemed to be in the induction of apoptosis in various systems, its future potential in cancer therapy justifies active research on its function in different developmental contexts.

A particularly intriguing thought would be to speculate that SFRP-4 could abrogate a survival mechanism in breast cancer stem cells, assuming both that such cell types indeed exist, and that they are "sufficiently" similar to mammary alveolar progenitors in which β-catenin signalling is necessary for mammary development and pregnancy-induced proliferation [reviewed in Ref. [54]].

## Conclusion

This study demonstrates that Wnt-3a treatment interferes with *in vitro *differentiation of mammary co-cultures to β-casein production in response to lactogenic hormones. Similarly, in another measure of differentiation, following Wnt-3a treatment mammary epithelial cells could be shown to up-regulate the *cyclin D1 *and *connexin-43 *genes while phenotypically they show increased transepithelial resistance across the cell monolayer. All these behavioural changes can be blocked in mammary epithelial cells expressing SFRP-4. A physical association between Wnt-3a and SFRP-4 was demonstrated. Thus, our data illustrate in an *in vitro *model a mechanism by which SFRP-4 can modulate a differentiation response to Wnt-3a.

## Methods

### Cell Cultures, Plasmid Constructs and Transfections

The mouse mammary epithelial Line 31E and fibroblast-like 30F were originally established from BALB/c mice in our laboratory [4] and were used to develop a co-culture model allowing functional differentiation of mammary cells to β-casein production *in vitro *[5]. These cells were routinely maintained in Eagle's Dulbecco-modified medium enriched with 10% foetal calf serum (EDM10). The co-cultures were established with densities of Line 31E to Line 30F in the ratio of 4:1. After culture for 48 hrs initially in EDM supplemented with EGF (10 ng/ml) and insulin (5 ug/ml), for lactogenic induction, cells were changed for 24 hrs to medium with 3% foetal calf serum, 5 ug/ml insulin, 1 ug/ml hydrocortisone and 5 ug/ml ovine prolactin. HEK 293 and L cells were employed for some experiments and were also maintained in EDM with 10% foetal calf serum, except that no EGF/insulin supplement was used.

Rat SFRP-4 [36] (Accession no. EMBL: AF 012891) cDNA was subcloned into the pcDNA6 (Invitrogen, Paisley, UK) plasmid containing a triple HA tag and into the pBabe^puro ^[55] retroviral vector. The Phoenix retroviral packaging cell line was the gift of Dr. Gerry Nolan [56] and was employed for preparation of infectious pBabe^puro ^SFRP-4. For infection of 30 F cells, a multiplicity of 0.5 to 1 was employed, followed after 48 hrs by transfer and selection with puromycin 2.5 μg/ml for a further 3 days.

The human Dkk1 gene was a generous gift of Dr C. Niehrs (DKFZ, Heidelberg). L cells were plated at a density of 10 × 10^5 ^per 10 cm dish, grown to 50–60% confluence and were then transfected with 20 μg of plasmid DNA with FuGENE 6 (Roche) according to the manufacturer's instructions. At 48 hrs post transfection, stimulation with purified Wnt-3a was performed and samples were collected for analysis. For stable transfectants, 48 hrs post transfection, cells were trypsinised and plated at 5 × 10^5 ^per 10 cm dish in the presence of Blasticidin (10 μg/ml) for the pcDNA6-SFRP-4(HA) construct.

### Purification of Wnt-3a

The preparation of partially-purified Wnt-3a was performed with a simplified protocol based on that described by Willert et al. [8]. Briefly, conditioned medium was harvested from L Wnt-3a cells (American Type Culture Collection) grown in EDM with 10% FCS at 2 day-intervals, cleared from cellular debris by centrifugation and stored at 4°C. 600 ml of such conditioned medium was adjusted to 1% Triton X-100, filtered through a 0.2-μm Millipore filter and applied to a Cibacron Blue F3G-A Sepharose 6 Fast Flow (Amersham Biosciences, Little Chalfont, England) column (bed volume of 50 ml), which had been previously equilibrated in binding buffer (150 mM KCl, 20 mM Tris-HCl pH 7.5, 1% Triton X-100). The column was then washed, first with 150 ml of binding buffer and then with 50 ml of binding buffer containing CHAPS instead of Triton X-100. Bound proteins were then eluted using 80 ml elution buffer (1.5 M KCl, 20 mM Tris-HCl pH 7.5, 1% CHAPS) and collected in 4 ml fractions. An aliquot of each fraction was then adjusted to 1 × SDS-PAGE sample buffer and analysed by SDS-PAGE followed by immunoblotting and detection with affinity-purified Wnt-3a-specific antibody. A second aliquot was electrophoresed and the bands detected with silver staining. The Wnt-3a elution peak was very broad according to the immunoblot. The corresponding band visible with silver staining was apparent. The same procedure was applied to conditioned medium from L cells as a control, but the purified product did not exhibit the Wnt-3a band in the same elution profile region and no Wnt-like stimulatory effect was seen in bio-assays. The enrichment obtained for Wnt-3a was approximately 70-fold. The eluted Wnt-3a was dialyzed and stored at 4°C. Because it contained some residual CHAPS which was necessary for solubility, Wnt-3a was always added to cultures at 2.5 – 5 μl/ml EDM with 3% foetal calf serum. Control plates received the mock-purified material (also containing residual CHAPS after dialysis) from normal L cells.

### Treatment of Cells with Wnt-3a and Preparation of Lysates

For stimulation experiments involving purified Wnt-3a, recipient cells (L, C57MG and HEK293 cells) were grown to confluence for 72 hours in 5 cm cell culture dishes using 5 ml culture medium. Line 30F cells and the co-cultures of 31E with 30F (4:1 ratio) cells were grown for 48 hours in 5 ml cell culture medium, and for the final 24 hours replaced by culture medium containing 3% foetal calf serum. 2 ml of medium was collected, supplemented with purified Wnt-3a (2.5 – 5 μl per ml EDM) or mock-purified conditioned medium from L cells (control) and re-applied to the cells after removing the remaining 3 ml of culture medium.

For determination of cytosolic β-catenin, cytosolic extracts were prepared after swelling in Hypotonic Lysis Buffer from confluent cultures on 5 cm plates as follows. Plates were incubated for 10 min on ice in 0.5 ml of Hypotonic Lysis Buffer [4 mM Na_2_HPO_4_, 20 mM KH_2_PO_4_, 3 mM MgCl_2_, 10% sucrose, and 1 mM 2-mercaptoethanol, pH 7.5 supplemented with 5 μg each aprotinin and leupeptin, 1 mM each β-glycerophosphate, 6-aminohexanoic acid, NaF, Na-orthovanadate and phenyl-methyl sulfonyl fluoride (PMSF)]. Following gentle Dounce homogenisation of swollen cells with 20 strokes, samples were centrifuged at 20'000 × *g *for 15 minutes and the resulting supernatant was designated the cytosolic fraction. After determining the protein concentration using the Bradford-based Bio-Rad Protein Assay (Bio-Rad, Hercules, CA, USA), the cytosolic lysates were diluted to 1× in SDS-PAGE sample buffer (80 mM Tris (pH 6.8), 2% Na dodecylsulfate (SDS), 10% glycerol, 2% 2-mercaptoethanol and 0.02% bromphenol blue) and boiled for 2 minutes.

Total cytoplasmic lysates containing both cytosolic and cell membrane proteins were obtained using a Triton X-100 containing Lysis Buffer. Briefly, plates were washed with Dulbecco's phosphate buffered saline (PBS) without Ca^2+^/Mg^2+ ^and incubated at 4°C for 10 min in 1 ml of Triton X-100 Lysis Buffer (PBS with 1% Triton X-100, 0.25% Na-deoxycholate, 1 ug/ml each leupeptin and aprotinin, 1 mM each EDTA, EGTA, β-glycerophosphate, 6-aminohexanoic acid, NaF, sodium-orthovanadate and PMSF, which was added to mM before use. Following centrifugation at 20'000 × *g *for 10 min the supernatants were collected. After determination of protein concentration using the Bradford-based Bio-Rad Protein Assay (as above), the lysates were diluted to 1× SDS-PAGE sample buffer, boiled and electrophoresed on 7.5% or 10% polyacrylamide gels.

Total Cell Lysates (TCL) were collected by lysing cells directly with 1× SDS-PAGE sample buffer (500 μl for a 5 cm cell culture dish) and boiling for 5 minutes.

### Immunoprecipitation Experiments

For immunoprecipitation of SFRP-4, 4 ml of a 72 hour conditioned medium from HEK293 SFRP-4(HA tagged) cells was centrifuged at 10'000 × g for 20 minutes. The cleared supernatant was then collected, mixed 1:1 with Immunoprecipitation Buffer (Dulbecco PBS without Ca^2+^/Mg^2+^containing 0.25% Triton X-100) and a rabbit anti-HA tag antibody (Santa Cruz, CA, USA) was added. This was then incubated at 4°C on a rotator for 5 hours; thereafter, 25 μl of protein G Sepharose beads (Amersham Pharmacia Biotech) were added for overnight incubation. The immunoprecipitated complexes were washed three times in Immunoprecipitation Buffer. SDS sample buffer (25 μl) was added, samples were boiled and analysed following electrophoresis by Western blot.

### SDS-PAGE

Electrophoretic separation of proteins was performed by discontinuous (Laemmli) SDS-PAGE using the Mini-PROTEAN^® ^II electrophoresis cell (Bio-Rad) with an electrophoretic separation length of 7 cm. For immunoblot analysis of Dvl-3, 7.5% polyacrylamide gels were used; otherwise 10% polyacrylamide gels were poured using research-grade 29:1-Acrylamide/Bisacrylamide (Serva, Heidelberg, Germany). To ensure that equal amounts of protein were loaded, protein concentration was determined by using a Bradford-based assay (Bio-Rad Protein Assay) and/or estimated by Coomassie staining of gels run in parallel and confirmed by Ponceau S staining of Western blot membranes.

### Immunoblot analysis

For Western Blot analysis, proteins were separated by SDS-PAGE as described above and transferred onto nitrocellulose membranes (Schleicher & Schuell Inc., Dassel, Germany) using a semidry electroblotter (Bio-Rad). After transfer, the membranes were incubated for 1 hour in blocking solution (20 mM Tris pH 7.6, 140 mM NaCl, 0.1% Tween-20, 5% w/v non-fat dry milk) and then washed three times for 5 min with Tris-buffered saline containing Tween-20 (TBS-T: 20 mM Tris pH 7.6, 140 mM NaCl, 0.05% Tween-20). Primary antibodies were diluted 1:1000 with TBS-T containing 5% w/v BSA and the membranes were incubated overnight at 4°C with gentle agitation. After three 5 min-washes with TBS-T, the membranes were incubated for 1 h at room temperature in blocking buffer containing the secondary antibody diluted 1:1000. After washing the membranes three more times for 5 min with TBS-T, the immunoreactive bands were visualised by chemiluminescence (using SuperSignal^® ^West Pico Chemiluminescent Substrate, Pierce, Rockford, IL, USA) and by exposing the membranes with a CCD camera system (Lumi-Imager™, Roche Diagnostics, Rotkreuz, Switzerland). In the case of total Akt, after immunodetection of Ser473-phosphorylated Akt as described above, the membrane was stripped by incubation for 30 min at 50°C with gentle agitation in stripping buffer (62.5 mM Tris-HCl pH 6.7, 100 mM 2-mercaptoethanol, 2% SDS), washed twice for 10 min with TBS-T, blocked for 1 hour with blocking solution and reprobed for total Akt.

### Antibodies

Polyclonal antibodies against β-catenin and Dvl-3 were raised in rabbits in our laboratory. In brief, the antigens were expressed in *E. coli *using the inducible pGex expression system (Amersham Biosciences) and purified by preparative SDS-PAGE. Rabbits were immunised repeatedly with 500 μg of antigen. For affinity purification, the sera were incubated with antigen coupled to Affigel-10 (Bio-Rad) and specific antibodies then eluted using 100 mM glycine buffer (pH 2.5), neutralized with 0.1 × volume 1 M Tris pH 8.0 after collection of the fraction. Finally, peak fractions were identified, pooled and dialyzed against PBS at pH 7.5. The specificity of the antibodies was confirmed by immunoblotting of *in vitro *translation products with the corresponding antibody. The TNT^® ^Coupled Reticulocyte Lysate System (Promega, Madison, USA) was used for the *in vitro *synthesis of β-catenin, and Dvl-3 proteins, according to the manufacturer's instructions.

A polyclonal rabbit antibody against mouse β-casein was generously provided by Dr. Ernst Reichmann (Pediatric Surgery Research, University Hospital, Zurich, Switzerland). The remaining antibodies were obtained from commercial sources: the mouse monoclonal antibody against cyclin D1 (sc-2004) and the rabbit polyclonal anti-HA antibody were purchased from Santa Cruz Biotechnology, Inc. (Santa Cruz, CA, USA), the mouse monoclonal β-actin antibody was from Sigma-Aldrich, the anti-mouse- and anti-rabbit-IgG-HRP-linked antibodies were from Amersham Biosciences (Buckinghamshire, UK), and, finally, the mouse monoclonal anti-HA antibody, the anti-phospho-ser473 Akt, the anti-phospho-thr308 Akt and the anti-Akt antibodies as well as the anti-phospho-Ser9/21 GSK3 antibodies were from Cell Signalling Technology (Beverly, MA, USA).

### Isolation of RNA and Northern blotting

Total RNA was prepared from cells using the guanidinium-thiocyanate extraction method described by Chomczynski and Sacchi [57]. 5 μg of polyA^+ ^RNA was denatured with glyoxal, electrophoresed on a 1% agarose gel and blotted to nitrocellulose. Equal loading was controlled by examination of bands formed by residual ribosomal RNA following staining with acridine orange, immediately prior to blotting as previously described [42]. Probes for hybridization were prepared using the Random Primed Labelling Kit (Roche) including γ^32^P-dCTP (800 Ci/mM; Amersham International, Little Chalfont, England) in the reaction and hybridization was carried out as previously described [42].

### *In Vitro *Differentiation Assays

In order to induce functional *in vitro *differentiation of Line 31E mouse mammary epithelial cells and Line 30F cells, co-cultures were prepared as previously described [5] with densities of Line 31E to Line 30F in the ratio of 4:1. After culture for 48 hrs initially in medium supplemented with EGF (10 ng/ml), cells were changed for 24 hrs to EDM with 3% foetal calf serum, 5 ug/ml insulin, 1 ug/ml hydrocortisone and 5 ug/ml ovine prolactin. Thereafter, supernatants were taken and cells were lysed using Triton X-100 Lysis Buffer for determination of β-casein in Western blots or with guanidine thiocyanate buffer, pH 4.0 for isolation of RNA.

For determination of the electrical resistance across the monolayers of pure cultures of Line 31E cells parallel cultures were grown to confluence on cellulose nitrate filter inserts (diameter 2.2 cm) in 6-well plates as previously described [5]. Resistance measurements were obtained with the Millipore (Bedford, MA., USA) Millicell-ERS instrument, essentially a milli-volt/ohmmeter that provides an alternating voltage source to minimise cell damage. Results were expressed as mean values for Ohms × cm^2 ^+/- S.E.M. taken on parallel inserts (n = 5).

## Competing interests

The authors declare that they have no competing interests.

## Authors' contributions

In partial fulfillment of the requirements for the PhD in the Faculty of Natural Science, University of Berne, TC and FB performed all experiments directed to measuring phosphorylation and other signalling intermediates, β-catenin stabilization and physical association between SFRP-4 and Wnt-3a. ML and SS prepared polyclonal rabbit anti-SFRP-4, performed the cloning necessary for antigen preparation and carried out Northern blot experiments. RF and AD directed the research and carried out studies on the influence of Wnt-3a on mammary epithelial cell differentiation including the measurements of transepithelial resistance. All authors read and approved the final manuscript.
